# Objective Disease Monitoring Strategies from a Tertiary Inflammatory Bowel Disease Center in Hungary

**DOI:** 10.5152/tjg.2023.22339

**Published:** 2023-05-01

**Authors:** Livia Lontai, Zsuzsanna Kürti, Lorant Gonczi, Nóra Komlódi, Fruzsina Balogh, Ákos Iliás, Peter L. Lakatos

**Affiliations:** 1 Department of Internal Medicine and Oncology, Semmelweis University, Budapest, Hungary; 2 Department of Gastroenterology, McGill University Health Centre, Montreal, Quebec, Canada

**Keywords:** Biological therapy,, clinical decision-making,, inflammatory bowel diseases,, therapeutics,, treatment outcome,, treatment switching

## Abstract

**Background:**

Emerging data suggest that a treat-to-target approach and early therapeutic intervention using regular objective disease assessment leads to improved outcomes. Our aim was to evaluate the value of objective disease monitoring during regular follow-up in a single tertiary inflammatory bowel disease center.

**Methods:**

Consecutive inflammatory bowel disease patients (n = 161, Crohn’s disease: 118/ulcerative colitis: 43; biological therapy: 70%) were included and followed up for 1 year between January and December 2018. Data on clinical disease activity, biomarkers, endoscopy, imaging, outpatient visits, treatment optimization, hospitalization, and surgery were collected. We compared the monitoring strategy according to the clinical activity (remission/flare/post-flare/continuous activity) every 3 months (assessment period).

**Results:**

In total, n = 644 assessment periods were evaluated. Biomarkers were evaluated in 82.9%-83.9% of patients in each assessment period regardless of clinical activity. Colonoscopy was more frequently performed in active disease (flare/continuous disease activity: 21.1%/18.9% vs. clinical remission: 10.1% per assessment period). Magnetic resonance imaging was performed in 7.7%-16.7%/period in Crohn’s disease patients, while the use of computed tomography was low (2.4%/period) and mainly performed in active disease. Treatment optimization was more frequent in patients with active disease (biological start/dose optimization: 31.1%/33.8%/period, steroid start: 13.2%/period). Patients with continuous activity (2.62), flare (2.45), and post-flare (2.05) had higher mean patient visit counts compared to remission (1.68/period).

**Conclusions:**

Objective monitoring strategy was applied with routine assessment of clinical activity and biomarkers. Fast-track colonoscopic evaluations were adapted to the clinical stage of the disease while screening colonoscopies and magnetic resonance imaging were frequently used. Objective monitoring resulted in the early optimization of medical therapy and frequent specialist follow-up visits.

**Main Points**

- Objective monitoring strategy was applied in this referral inflammatory bowel disease (IBD) cohort with a routine assessment of clinical activity and biomarkers (complete blood count and C-reactive protein), irrespective of symptomatic disease activity
- The use of endoscopy and magnetic resonance imaging was relatively high, while computed tomography was reserved for acute events, and access to expert IBD ultrasound was relatively low.
- Objective monitoring resulted in early optimization of medical therapy and high number of specialist follow-up visits.

## Introduction

Inflammatory bowel diseases (IBD) are chronic and progressive diseases that significantly affect patients’ quality of life and in the long term lead to complications and disability. Therefore, it is crucial to optimize the quality of care to improve long-term outcomes. Quality of care indicators have been developed for IBD, and they are suggested to be integrated into daily clinical practice.^1^

Symptomatic disease control is suboptimal since there is a discordance between symptoms and objective measures of disease severity. This led to the development of “treat-to-target” (T2T) strategy, with the aim to achieve objective disease control (clinical, biochemical, and endoscopical/histological) beyond symptomatic remission using a timely monitoring strategy. According to the Selecting Therapeutic Targets in Inflammatory Bowel Disease I (STRIDE-I) recommendations, the combination of clinical/symptomatic remission (patient-reported outcomes, clinical disease activity scores) and endoscopic remission is the main target in IBD.^2^ Clinical disease activity should be re-assessed every 3 months in active disease. Endoscopic disease activity should be monitored every 6 to 9 months in active disease. Intervals of optimal monitoring are less clear in patients in disease control. Biomarkers, including C-reactive protein (CRP) and fecal calprotectin (FCAL), were adjunctive measures for monitoring in the STRIDE-I recommendations but were re-classified as treatment targets in the recent STRIDE-II recommendations.^3^ This means in the clinical practice that an elevated CRP or FCAL should prompt further evaluation and/or optimization of the therapy.

Emerging data suggest that a T2T approach and early therapeutic intervention using regular objective disease assessment can lead to improved outcomes. The prospective randomized CALM study demonstrated how tight control, whereby treatment decisions are based on close monitoring of inflammatory biomarkers and clinical symptoms, leads to improvements in endoscopic and clinical outcomes compared to conventional care based on symptoms alone.^4^ Of note, patients who achieved deep remission at 1 year had improved long-term outcomes over a median 3 years of follow-up, regardless of tight management or conventional management strategy.^5^ Recently, a paper from Canada suggested that early objective complex assessment at 3 months resulted in earlier dose optimization, improved clinical outcomes, and higher clinical remission at 12 months.^6^

Nevertheless, real-world data on the assessment of monitoring practices in IBD patients are still limited.^7^ Many factors may limit the applicability of T2T in clinical practice. For instance, the adherence to serial FCAL testing was low in several real-world cohorts.^6,8^ Other IBD centers have challenges in implementing this approach systematically because of the physicians’ attitudes or resource-related factors such as the cost of FCAL measurement or endoscopy.^9^

Our aim was to assess the appropriateness of monitoring strategy and treatment optimization practices in IBD in the everyday clinical practice in a single tertiary IBD center from Hungary.

## Materials and Methods

Consecutive IBD patients presenting in our tertiary referral IBD center were included retrospectively between January and December 2018. Baseline demographics and clinical characteristics data (age, date of diagnosis, location, and behavior or extent of disease, current and failed medications, prior surgeries, and other medical conditions) were collected and comprehensively reviewed. Disease location and behavior were classified according to Montreal classification.^10^ Patients were followed up for a period of 1 year. The follow-up was divided into 3-month periods (assessment periods), as this is the suggested follow-up frequency in patients with active disease in the STRIDE recommendations.^2^

In every assessment period, patients were evaluated for clinical disease activity using Crohn’s Disease Activity Index (CDAI) in Crohn’s disease (CD) and partial Mayo Score (pMayo) in ulcerative colitis (UC), in accordance with the current Hungarian regulation. In addition, data on biomarkers (complete blood count (CBC), CRP, FCAL test) were systematically collected if available. In addition, stool culture, endoscopic data (colonoscopy/sigmoidoscopy), imaging results [computed tomography (CT)/magnetic resonance imaging (MRI) scans, abdominal ultrasound (US)], total number of visits, hospitalization or surgery rates, and change(s) in medical therapy were collected. Magnetic resonance imaging scans were either MR enterographies in CD patients with small bowel disease or penetrating/stenosing disease phenotype or pelvic MRI for the assessment of perianal fistulas.

In every assessment period, patients were classified into 4 categories according to the actual clinical (i.e., symptomatic) disease activity: remission, flare, post-flare, or continuous activity. These categories were defined based on the CDAI/pMayo score following the recommendations of the European Crohn’s and Colitis Organisation (ECCO) guidelines; CD remission is defined as a CDAI <150, or no fistula drainage, while active disease as CDAI >150 or active perianal fistulas.^11^ As for UC, remission was defined as pMayo ≤2, active UC as pMayo ≥3, or a change in medical therapy.^12^ Disease flare was considered, when symptomatically active disease was detected in one assessment period and remission in the previous one. Post-flare disease activity stage was defined when clinical disease activity showed remission, but in the previous 3-month period, symptomatically active disease was detected. In our analysis, we compared the monitoring strategy stratified by the clinical/symptomatic disease activity in each assessment period (remission/flare/post-flare/continuous activity).

The study complies with the principles of the Declaration of Helsinki. The study protocol was approved by the Semmelweis University Regional and Institutional Committee of Science and Research Ethics (SE TUKEB 142/2010). Informed consent was not required due to the retrospective nature of the study.

### Statistical Methods

Statistical analysis was performed using the Statistical Package for the Social Sciences software v. 20.0 (IBM Corp.; Armonk, NY, USA). Descriptive statistics were calculated and variables were tested for normality using Shapiro–Wilk’s W-test. The Chi-square test and Chi-square test with Yates correction were used to describe associations between categorical variables. For categorical data, frequency distributions were analyzed, and for continuous variables, medians and interquartile ranges were calculated. The Chi-square test was used to evaluate differences within subgroups of patients. *P* <.05 was regarded as statistically significant.

## Results

### Patient Characteristics

A total of 161 patients were included (CD: 118/UC: 43; male: 56%), with predominantly moderate-to-severe disease phenotype. Seventy percent of the patients were on biological therapy and 20% of patients have already received prior biological therapy with inadequate response. Patients’ demographic data are presented in Table 1.

### Frequency of Clinical Visits and Clinical Disease Activity

Of the 161 patients, 114 patients (70.8%) had at least 1 follow-up visit in every 3-month assessment period. Consequently, out of the 644 (n = 644) potential assessment periods, 554 (n = 554) periods with a doctor–patient visit were evaluated for monitoring strategy and interventions. Every 3-month period was classified into 4 categories according to the actual clinical (i.e., symptomatic) disease activity: remission n = 316 (57%); continuous activity n = 106 (19%); post-flare n = 61 (11%); and flare n = 71 (13%).

The CDAI scores were determined in almost all patients. Mean scores were 231.7 (SD: 72.4) in patients with continuous disease activity and 220.7 (SD: 77.7) in patients with flare, whereas it was 93.1 (SD: 35.1) in patients with post-flare and 62.4 (SD: 36.8) in patients in remission in CD. In UC, the use of the pMayo score was universal, the mean score was 4.8 (SD: 1.6) and 4.03 (SD: 1.1) in patients with continuous activity and flare, and 0.9 (SD: 0.7) and 1.1 (SD: 0.8) in patients with post-flare and remission (Table 2).

The mean number of clinical follow-up visits per assessment period was 1.68 for patients in remission, 2.05 for patients in post-flare, 2.45 for patients in disease flare, and 2.62 for patients with continuous disease activity stage. In a sensitivity analysis of patients not receiving biological therapy—representing patients with a less severe disease phenotype—the number of clinical follow-up visits was not lower compared to patients on biological therapy (1.5 for patients in remission, 2.0 for patients in post-flare, 2.2 for patients in disease flare, and 3.6 for patients with continuous disease activity per assessment period).

### Objective Monitoring Strategy (Biomarkers, Endoscopy, Imaging)

**Figure 1.**
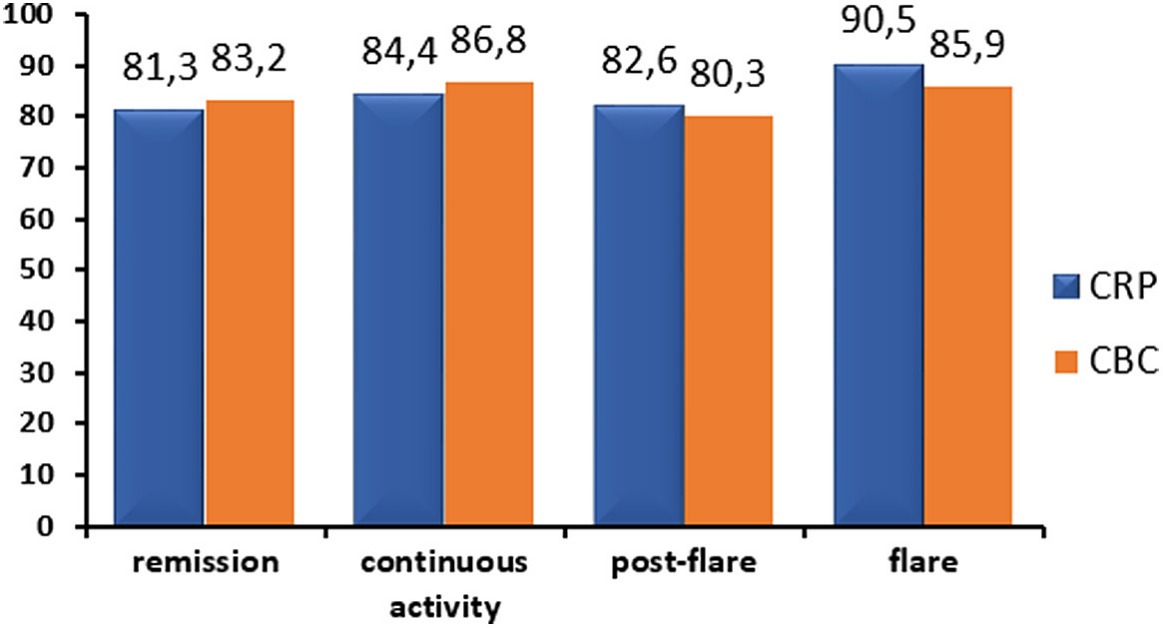
Frequency of complete blood count and C-reactive protein tests in patients according to the clinical disease activity per assessment period.

Biomarker assessment (CBC and CRP) was performed in 82.9% and 83.9% of the patients in each assessment period. Complete blood count and/or CRP tests exceeded 80% in each period, regardless of clinical disease activity. Patients with flare had serum biomarker evaluation over 90% in the trimonthly periods (Figure 1).

In contrast, FCAL (not reimbursed in Hungary) and stool culture/*C. difficile* tests were performed altogether at a low rate. Fecal calprotectin test was used only in a minority of the patients for disease monitoring (UC: 3.5%, CD: 1.2% per assessment periods). Stool culture/*C. difficile* tests were used in patients with continuous activity or flare. Stool culture and *C. difficile* stool tests were performed in 17.2% and 10.3% of UC patients with flare and in 9.5% and 7.1% of CD patients with flare and ~0% in remission per assessment period.

**Figure 2.**
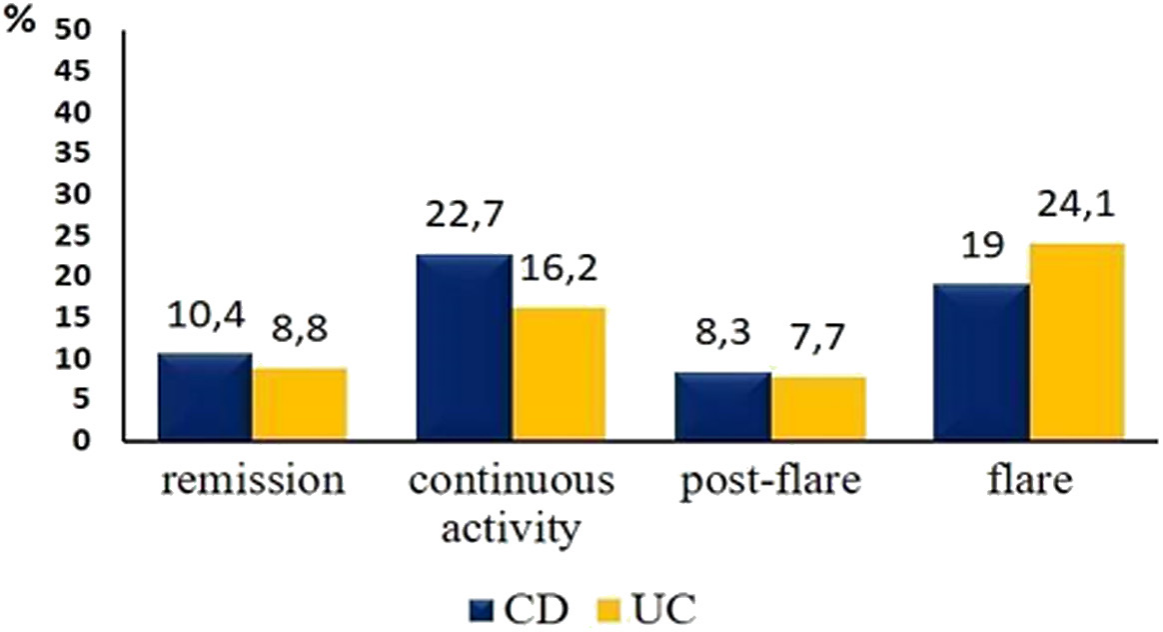
Frequency of colonoscopy/flexible sigmoidoscopy according to the clinical disease activity per assessment period.

Endoscopic evaluation was frequent. Colonoscopy was performed more often in a given 3-month assessment period in patients with flare or continuous disease activity (21.1% and 18.9%); however, colonoscopy was performed in 10.1% of the periods with clinical remission for monitoring/screening purposes. In a sub-analysis of UC patients, 24.1% of the patients presenting with disease flare had a colonoscopy in the given assessment period. In addition, 7.7% of UC patients had flexible sigmoidoscopy in an assessment period if they were in a post-flare disease activity stage. Among CD and UC patients with continuous disease activity, 22.7% and 16.2% had colonoscopy or flexible sigmoidoscopy in an assessment period (Figure 2).

**Figure 3.**
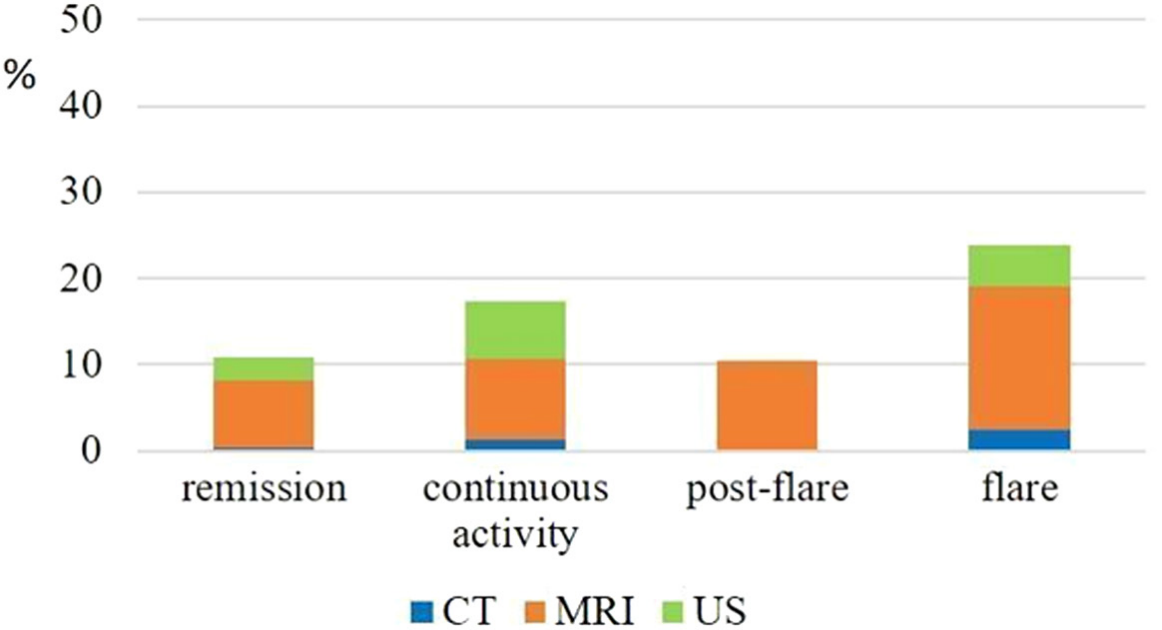
Frequency of abdominal imaging according to the clinical disease activity per assessment period in Crohn’s disease.

Magnetic resonance imaging was performed frequently particularly in CD patients, regardless of clinical activity (remission: 7.7%, flare: 16.7%, post-flare: 10.4%, continuous activity: 9.3% per assessment period). In contrast, CT scans were performed at a low rate mainly in patients with disease flare (2.4% in CD and 3.4% in UC per assessment period). Abdominal US was performed mainly in CD patients with flare (4.8%) or continuous activity (6.7% per assessment period), but the use of this modality was altogether low (Figure 3).

### Therapy Modifications and Outcomes

In the total IBD cohort, initiation or dose optimization of systemic corticosteroids was performed in 9.4%/3.8% of patients with continuous disease activity and in 18.3%/4.2% of patients with disease flare in a given trimonthly assessment period. Start or dose optimization of biological therapy was needed in 24.5%/6.6% and 21.1%/12.7% in the continuously active or flare patients in a given assessment period. The initiation or dose optimization of immunomodulators (mainly azathioprine) was performed in 5.7%/1.9% of patients with continuous disease activity and 5.6%/2.8% in patients with disease flare in the total IBD cohort. In a sub-analysis of patients already on biological therapy, additional immunosuppressive medication start was 4.2% and 3.8% per assessment period in continuously active and flare patients, respectively.

**Figure 4.**
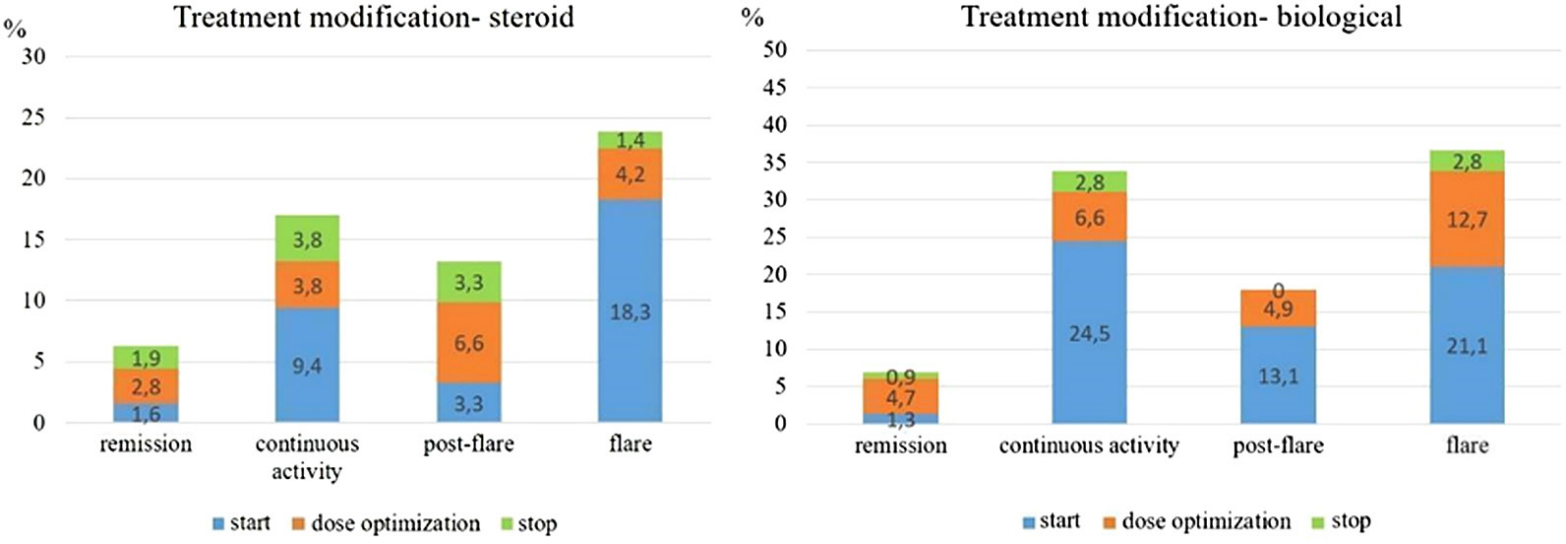
Frequency of treatment modifications (steroid and biological therapy) according to the disease activity stage per assessment periods in the total inflammatory bowel disease cohort.

In patients with clinical remission, initiation or dose optimization of steroid, immunomodulator, and biological therapy was rare, 1.6%/2.8%, 1.9%/3.2%, and 1.3%/4.7% per assessment period and this was based on the results of the objective disease activity measures (biomarkers/endoscopy). For detailed frequencies of treatment modifications (steroid and biological therapy) stratified by the symptomatic disease activity stage, see Figure 4.

The number of hospitalizations and surgical procedures was relatively low in the cohort. In the total cohort, the all-cause hospitalization rate was 13/118 (11%) in CD patients and 8/43 (18.6%) in UC patients during the total 1-year follow-up. Among CD patients hospitalized, 8 out of 13 patients presented with a disease flare, while 6/8 UC patients hospitalized were due to disease flare. Overall surgery rates during the 1-year follow-up were 4/118 (3.4%) in CD patients (2 patients with abdominal respective surgery and 2 patients with perianal surgery), and 2/43 (4.6%) in UC patients (1 colectomy and 1 rectal stump extirpation).

## Discussion

The major finding of the present study was that our center adopted an objective monitoring strategy in this referral IBD cohort with serial assessment of clinical activity, biomarkers, and frequent use of colonoscopy and MRI—both in patients in remission and experiencing flare. The frequency of specialist follow-up visits was high, irrespective of disease activity state. Of note, a large proportion of the patients in our study presented severe disease course and were treated with biological therapy. These objective monitoring strategies resulted in early treatment optimization or change of medical therapy in patients with flare or continuous disease activity.

Some years ago, the CALM study4 provided evidence that serial, objective disease monitoring is associated with superior outcomes in patients with moderate-to-severe CD. The same strategy is increasingly applied to patients with UC, especially in patients treated in IBD centers. However, “real-world” clinical data on the benefit of the universal use of stringent objective assessment in IBD centers, or more interestingly in mild IBD patients followed up by general practitioners (GPs) or community gastroenterologists, are still very limited.

Until now, relatively few studies assessed the appropriateness of patient monitoring in real-world cohorts from IBD centers or the general practice. One of the early studies was published from our center. Gonczi et al^13^ reported structural and procedural quality of care indicators, including data on the frequency of disease flares, access to IBD specialist physician, and endoscopic/imaging procedures in a referral IBD center. During an observational period of 2 years (2014-2016) patients with flares (CD/UC: 50.6/54.6%) were seen by a specialist at the IBD clinic within a median waiting time of 1 day with same day laboratory assessment in almost all cases. However, follow-up strategy and adherence to serial monitoring strategy with clinical and biomarker assessment were not evaluated in this article. In contrast, in the present study, frequent, timely patient access to specialist physicians was offered, and the mean number of clinical follow-up visits ranged from 1.68 per study assessment period (per 3 months) in patients in remission to 2.62 per 3 months in patients with continuous disease activity. Moreover, the use of clinical scores, CDAI and pMayo, was universal.

More recent “real-world” data on disease assessment and monitoring strategies come from a Canadian academic referral IBD center. In the study by Reinglas et al^14^ from the McGill IBD center, 1357 patients were included in a retrospective analysis. Authors reported that during a 2-year observation period (2014-2016) colonoscopy was performed in 79% of the total patient population, while imaging was available in 21.4%, 23.2%, and 17.3% of the patients using MRI, CT, and abdominal US, respectively. Over a 6-month evaluation period, CRP and FCAL were measured as 78% and 37%, respectively. However, the physicians’ clinical follow-up strategy was not assessed.

Another study by the same working group focused on adherence to objective disease monitoring strategies (serial monitoring of clinical symptoms and biomarkers) in patients treated with biologicals.^6^ Authors collected data on 428 consecutive IBD patients newly started on adalimumab therapy. Clinical assessment, CRP, and FCAL testing frequency were 95.5%, 70.6%, and 25.4% in CD patients and 94.3%, 64%, and 33.3% in UC patients, respectively, after 3 months of therapy start. Adherence to clinical assessment was similar at 6 months and 1 year after a biological start; however, CRP and FCAL testing decreased. Combined adherence to clinical and biomarker monitoring up to 6 months was also different between the academic center and another affiliated hospital (47.2% vs. 32.3%, *P* = 0.014).

Finally, an Australian multicenter retrospective study aimed to survey clinical and objective assessment of disease activity (endoscopy, histology, and/or biomarkers) in 246 patients with UC, where the benefit of a T2T approach is less proven.^9^ Over a course of 18 months, endoscopic assessment was performed in 89% of all patients, while FCAL testing was only available for 9% of the population—authors refer to financial/reimbursement issues with FCAL testing, similar to our situation in Hungary. Of the 61 practicing gastroenterologists surveyed, 80% of respondents had heard of the “Treat-to-Target” concept in UC, but only 64% considered it relevant to local clinical practice.

In our present study, in a predominantly CD cohort, biomarker assessment (CBC and CRP) was performed in 82.9% and 83.9% of patients in each 3-month assessment period regardless of clinical activity, which may be considered very tight monitoring of biomarkers, given that other studies report somewhat lower rates after 3 months in patients with a new biological therapy start.^6^ Fecal calprotectin was performed at a low rate in our patients; however, the test is not reimbursed in Hungary.

Comparing data regarding frequency of endoscopy and/or radiological imaging across studies is quite challenging because of the different methodologies and patient cohort characteristics of the studies. Our study may present a unique approach by trying to determine the use and instruments of objective disease monitoring, stratified by disease activity in a given quarter-year period. In our analysis, in a quarter-year period where patients had a recorded symptomatic disease flare, a colonoscopy was performed within 3 months in ~25% of UC patients. The corresponding rate for patients in remission was ~10% for CD and ~9% for UC patients in a given quarter-year period. Extrapolating these data would mean that approximately 40% of the patients in symptomatic remission undergo endoscopic evaluation in a given calendar year, which is considerably high. The reasons for this high rate are multiple. First, because of the known discordance between symptomatic and endoscopic activity, especially in CD patients, several endoscopies were prompted based on biomarkers. The STRIDE recommendation also states that CD and UC patients should be assessed with endoscopy after a period of 6-9 and 3-6 months, respectively, after therapy initiation or change, regardless of symptomatic activity. Lastly, our patient population represents largely severe disease phenotype, with over 80% of colonic involvement in CD patients, which makes frequent colorectal cancer screenings justified in those patients as well.

Magnetic resonance imaging was also used frequently in our center, and besides the important role in sub-acute evaluation of patients presenting with active disease, a considerable number of MRI examinations were performed in patients with remission, for follow-up purposes. By assuming that very few patients may have had multiple MRI scans within the same year, more than one-third of the study population had an MRI during the 1-year study period. The value of fast-track MRI in clinical decision-making and earlier optimization in a patient population with predominantly complex phenotype (structuring and fistulizing Crohn’s disease) has been previously reported by our study group.^15^ In contrast, the use of US as a point of care test was less frequent, compared to our earlier study, which reflects more easy access to MR and increasing waiting times for US.^13^ In the study by Gonczi et al^13^ a total of 86.7% of CD patients had any imaging evaluation in the period of 2014-2016 (US: 49.7%, CT: 5.6%, MRI: 39.3%), and 35.9% of UC patients underwent abdominal US.

Of note, in Hungary, the serial objective assessment of patients receiving biological therapy including monitoring of clinical scores (CDAI and pMayo), CRP, CBC every 3 months with yearly assessment of endoscopy (colonoscopy), or cross-sectional imaging is mandated also by the national public insurance bureau (NEAK) to measure patient benefit and to authorize ongoing maintenance therapy. Consequently, the monitoring of patients on biological therapy is harmonized throughout the country in IBD centers at least for patients receiving biological therapies.

Treatment optimization strategy was a further outcome measure in the present study. In the present cohort, in patients with severe clinically active disease, corticosteroids were started in ~20%, while biological therapy start or optimization was performed in ~30%/30% of the patients. A change in medical therapy was much lower in patients in symptomatic remission; however, around ~15% of these patients still underwent therapy optimization (corticosteroids, immunosuppressives, or biologicals), based on the results of objective disease activity measures (biomarkers/endoscopy). Similarly, in the study of Al Khoury et al^6^ optimization of the therapy in patients with inadequate clinical response and abnormal CRP/FCAL levels was around 50% in every 3 months assessment period compared to ~10% therapy modification in patients with adequate clinical response and normal biomarkers (*P* < .001).

Monitoring “big ticket” disease outcomes, such as hospitalization and surgery rates, is important quality indicator for IBD centers. The hospitalization rate in the present cohort was relatively low altogether. Rates were highest in UC patients with a disease flare. The frequency of any surgical procedure was 2.9% in the total cohort per assessment period. In a Canadian tertiary IBD center,^14^ the need for surgery (4.3%) and hospitalization (7.6%) were relatively low, while 16.8% of patients needed an IBD-related ER visit within a 6-month study period. However, comparing hospitalization rates is challenging, partly because of differences in healthcare systems (availability of GPs or emergency departments (ED) to provide first-line care). A prospective study evaluated the effect of the implementation of a “rapid access clinic service” at a tertiary IBD center at McGill University and reported significantly improved access times and cost-savings by lower ED visit rates in patients having quick access to their treating team.^16^

An economic model has been used to evaluate the cost-effectiveness of a tight control strategy vs. conventional clinical management, using data from the CALM trial. This analysis indicated that cost benefits associated with increased remission rates, reduced hospitalizations, and improved quality of life outweighed the increased drug costs and additional tests.^17^

The strengths of the present study include harmonized mandatory monitoring strategy of patients treated with biologicals, including easy access to clinical visits offered to all IBD patients of the IBD center. Patients were consecutively included, regardless of disease severity and actual disease activity, to avoid selection bias.

The limitation of this article is the retrospective methodology. The use of FCAL was low, due to the lack of reimbursement by the national healthcare provider. As expected in an IBD center, the percentage of biological therapy was high in our patients, which makes our results representative for IBD centers but not community GI practices with less severe IBD populations. The access to and use of cross-sectional imaging modalities (e.g., US, CT, MRI) may also show large variations based on local availability.

In summary, objective monitoring strategy was applied in this referral IBD cohort with a routine assessment of clinical activity and biomarkers. Fast-track colonoscopic evaluations were adapted to the clinical stage of the disease while screening colonoscopies and MRI imaging for monitoring of therapeutic efficacy in severe CD patients were frequently used. Computed tomography was reserved for acute events, while the use of US was relatively low. Objective monitoring resulted in early optimization of medical therapy and is coupled with a substantially high number of specialist follow-up visits.

## Figures and Tables

**Table 1.** Baseline Patient Characteristics

	CD/UC (n = 118/43)
Sex (male/female)	90/71
Age (mean (SD), years)	38 (11.1)
Disease duration (median (IQR), years)	11 (7.5-16)
Age at diagnosis (Montreal-classification A1/A2/A3, %)	5/65/30
CD (n = 118); disease location (Montreal-classification L1/L2/L3/+L4, %)	10/18.3/62.5/8.4
CD (n = 118); disease behavior (Montreal-classification B1/B2/B3, %)	54.7/29.1/14.5
CD (n = 118); perianal disease (%)	60.8
UC (n = 43); disease extent (E1/E2/E3, %)	9.8/51.2/39.0
Maximal treatment step (5-ASA/systemic corticosteroids/azathioprine/aTNF/UST or VDZ, %)	1.2/5.0/13.0/73.3/7.5
Previous resective surgery (%)	24.8
Previous biological therapy (%)	20.5
Current immunosuppressive (azathiprine) therapy (%)	31.1
Current biological and combined biological + IS therapy (%)	70.2/15.5

5-ASA, 5-aminosalicilates; aTNF, anti-tumor necrosis factor-α; CD, Crohn’s disease; IS, immunosuppressive; IQR, inter-quartile region; Montreal classification (A, age; B, behavior; L, location; E, extent); SD, standard deviation; UC, ulcerative colitis; UST, ustekinumab; VDZ, vedolizumab.

**Table 2.** Mean Clinical Activity Scores of All 3 Months Assessment Periods Classified into 4 Categories of Symptomatic Remission, Continuous Activity, Flare, and Post-Flare (n = 554)

	Remission	Continuous activity	Post-flare	Flare
CD (number of assessment periods)	n = 259	n = 75	n = 48	n = 42
Mean CDAI (SD)	62.4 (36.8)	231.7 (72.4)	93.1 (35.1)	220.7 (77.7)
UC (number of assessment periods)	n = 57	n = 31	n = 13	n = 29
Mean pMayo (SD)	1.1 (0.8)	4.8 (1.6)	0.9 (0.7)	4.03 (1.1)

CD, Crohn’s disease; CDAI, Crohn’s Disease Activity Index; pMayo, partial Mayo Score; SD, standard deviation; UC, ulcerative colitis.
